# The impact of non-responders on health and lifestyle outcomes in an intervention study

**DOI:** 10.1186/1756-0500-7-632

**Published:** 2014-09-11

**Authors:** Elsebeth Hansen, Kirsten Fonager, Kirsten S Freund, Jørgen Lous

**Affiliations:** Department of Social Medicine, Aalborg University Hospital, Havrevangen 1, DK-9000 Aalborg, Denmark; Department of Health Science and Technology, Faculty of Medicine, Alborg University, DK-9000 Aalborg, Denmark; General Practice, DK-9362 Gandrup, Denmark; Research Unit for General Practice, Institute of Public Health, University of Southern Denmark, DK-5000 Odense C, Denmark

**Keywords:** Health interview survey, Non-response, Primary care, Response bias, Telephone reminder

## Abstract

**Background:**

A randomized intervention study, “Preventive consultations for 20- to 40-year-old young adults”, investigated whether preventive consultations with a general practitioner could help young adults with multiple psychosocial and lifestyle problems to change health behavior. To optimize the response rate of questionnaires at 1 year post-intervention, the non-responders were reminded by telephone. The aim of this study was to examine potential selection bias induced by non-response by comparing responder and non-responder populations at baseline, and to examine the impact on outcomes by comparing initial respondents to respondents after telephone reminding.

**Method:**

Non-responders were compared with primary responders using logistic regression models that included socio-demographic factors, health-related factors, and variables related to the intervention study. In order to describe the impact of including responders after telephone reminding on the intervention’s effect on different health, resource, and lifestyle outcomes, we compared results in models including and excluding responders after telephone reminding.

**Results:**

Telephone contact raised the response by 10% from 316 (64%) to 364 (74%) among young adults with multiple problems. Being male was the only factor that significantly predicted non-response in the model after adjustment for other variables. The responders after telephone reminding tended to improve health and lifestyle more than the primary responders, but not significantly so. Although the additional responses did not change the estimates of the 1-year effect on health and lifestyle changes, it contributed to increased precision of the results.

**Conclusion:**

Even though the population of primary non-responders had to some degree a different composition than the primary responders, inclusion of responders after telephone reminding did not significantly change the estimates for effect at the 1-year follow-up; however, the additional responses increased the precision of the estimates.

**Trial registration:**

ClinicalTrials.gov: NCT01231256

## Background

In surveys, many efforts are taken to optimize response rate in order to render the results generalizable to populations of interest. The literature contains several suggestions regarding how to optimize response rates in surveys [1–7]. Many fanciful “tricks” have been described, such as attaching a pencil [2] or giving questionnaires a special color [1–3]. Lottery-style incentives had an effect on response rates to a postal health questionnaire in some studies and no effect in others [7]. The response rate might increase when a short questionnaire is used [4]. Sending a new questionnaire has been more effective than sending a reminder postcard [5]. Another study found that implementation of reminder letters and telephone contact had the most significant effect on response rates [6].

However, attempts to achieve a high response rate might not have the intended result. Recent studies have shown that although there were differences between groups of responders and non-responders, an increased response rate after reminders did not necessarily change significantly response patterns and study conclusions [8–11]. In a Norwegian population-based survey published in 2002, sending reminder letters and conducting a telephone follow-up made a small additional contribution to prevalence estimates, and the exposure-disease relation was small [11]. In a cross-sectional patient survey that examined patients’ perceptions of hospital care, the tendency to participate was negatively associated with the report of problems during hospitalization. Nevertheless, increasing participation from 30% to 70% had only a modest influence on the conclusions of the survey [9]. Comparing two different samples with different response rates from the same population yielded consistent estimates of exposure-outcome relationships [10]. However, increasing the response rate by issuing multiple reminders does not rule out the risk of non-response bias. Multiple reminders had a minor effect on response patterns and study conclusions in a Danish health survey, indicating that if differences do exist between responders and non-responders, multiple reminders would not solve non-responding bias [8].

Freund and Lous conducted a randomized intervention study entitled “Preventive consultations for 20- to 40-year-old young adults” from 1998 to 1999 [12, 13]. The intervention consisted of consultations with a general practitioner with the purpose of helping young adults with multiple psychosocial and lifestyle problems to change health behavior. One-year follow-up questionnaires were sent to the participants. After letter reminders were issued, they sought to increase the response rate by reminding via telephone, which is time-consuming.

The aim of this study was to examine potential selection bias induced by non-response by comparing responders and non-responders at baseline, and to examine the impact on outcomes by comparing the initial respondents to respondents after telephone reminding.

## Methods

A randomized intervention study was conducted from May 1998 to December 1999, entitled “Preventive consultations for 20- to 40-year-old young adults”; the results were described in 2002 and 2012 [12, 13]. The target group for the intervention study was the most disadvantaged socially, psychologically, and medically. Participants were selected by means of a problem-screening questionnaire, as described in an earlier study [12].

Informed written consent was obtained from all participants and included acceptance of later contact. The participants with seven or more problems were randomized to intervention (two preventive counseling sessions with a general practitioner) or to the control group. A total of 495 participants were randomized after they had completed a more extensive baseline questionnaire. From the baseline questionnaire, we obtained information on sociodemographic factors such as sex, age, civil status, education, and professional training, as well as information about self-rated health, health-related quality of life (SF-12), and how many times the participants had contacted the general practitioner in the last year.

One year later, all 495 participants were sent a follow-up questionnaire and up to two reminders by mail. To increase the response rate further, a physician tried to contact the remaining 179 (primary non-responders) by telephone. During the telephone reminder, they were asked to return the questionnaire, and were offered a new one if the original had been lost. Participants were also asked a few questions about why they had not returned the questionnaire, and finally they were asked about self-rated health.

Non-responders were compared with primary responders using logistic regression models including socio-demographic factors, health-related factors, and variables related to the intervention study. We described the impact of including responders after telephone reminding (responders included after they received a telephone call from a physician) on the intervention’s effect on different health, resource, and lifestyle outcomes by comparing the results in models including and excluding responders after telephone reminding.

Statistical tests were two-tailed, and P < 0.05 was considered significant. Stata version 11.2 (Stata Corp. 2009. Stata: Release 11. Statistically Software. College Station, TX: Stata Corp LP) was used for statistical analysis. The study was approved by the Danish Data Protection Agency (j.nr. 1997-1200-581).

## Results

**Figure 1:**
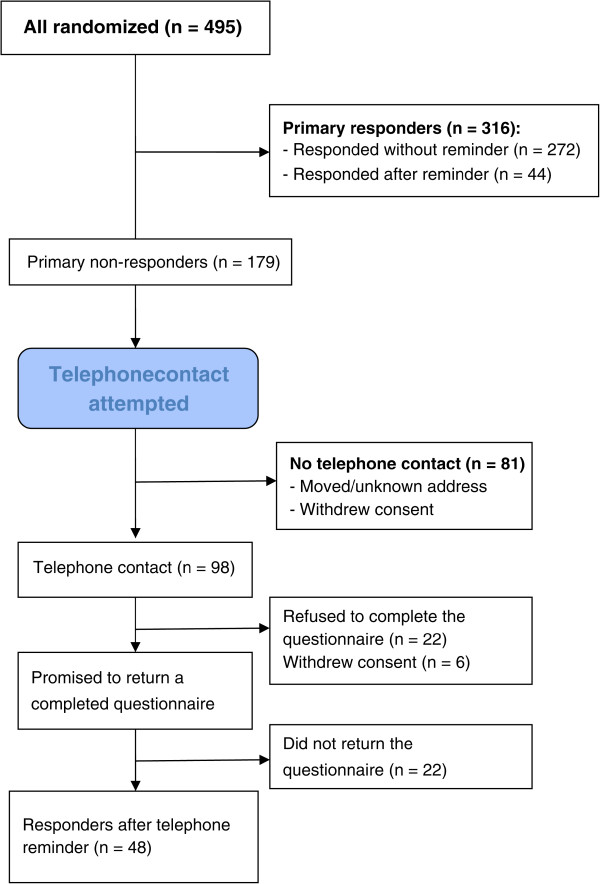
**Disposition of study participants.**

Of the 495 randomized patients, 272 (55%) returned the 1-year follow-up questionnaire without any reminder, and another 44 (9%) returned it after one or two reminding letters (Figure 1). Of the 179 (36%) primary non-responders, we managed to make telephone contact with 98 (20%). Of the 81 without contact, 12 had withdrawn their consent to participate in the study, and 69 did not answer up to five telephone calls, had an unidentifiable telephone number, or had moved to an unknown address. Of the 98 reminded by telephone, 70 (14% of the 495 randomized patients) promised to return a completed follow-up questionnaire, and 48 (10% of the randomized patients) actually did so. Thus, use of a telephone reminder raised the response rate from 64% to 74%.

During the telephone interview, participants gave different reasons for not returning the questionnaire. Many problems and lack of energy (n = 28) were frequent answers, although four respondents said that they no longer had any problems. Few considered the questions and questionnaires irrelevant 1 year later. Out of 98 possible, 80 patients were scored on a self-rated health scale (five degrees) during the telephone contact. Sixty percent said they had fair or bad health, compared with 49% in the primary responder group after 1 year. The difference of 11% was not statistically significant (95% CI: 0.7 to 22).

Possible predictors for non-response to the 1-year follow-up questionnaire are listed in Table 1. Being a man was the only factor that significantly predicted non-response in the crude and adjusted model. Although young age appeared to predict non-response in the crude model, in the adjusted models the association weakened and was no longer significant (Table 1). Smoking at baseline tended to predict non-response 1 year later; the association was stronger but not significant in the adjusted model.

**Table 1:** **Predictors of non-response to questionnaires sent 1 year after preventive health counseling in general practice**

		Total	Primary non-responders	Primary responders		
		*N = 495*	*N = 179*	*N = 316*		
		n	N *(%)*	N *(%)*	OR (95% CI)	OR (95% CI)*
Socio-demographic factors at baseline	Sex					
	Male	132	59 *(33%)*	73 *(23%)*	*1.64 (1.06-2.50)*	*1.69 (1.09-2.63)*
	Female	363	120	243	1	1
	Age					
	≤30 years	153	66 (37%)	87 *(28%)*	*1.51 (1.01-2.13)*	1.44 (0.95-2.20)
	>30 years	342	113	229	1	1
	Civil status					
	Single	143	56 *(31)*	87 *(28)*	1.17 (0.76-1.78)	1.03 (0.6-1.59)
	Cohabitant	347	123	224	1	1
	School education					
	Primary and lower secondary school	324	119 *(66%)*	205 *(65%)*	1.07 (0.72-1.61)	1.01 (0.66-1.54)
	Graduated	171	60	111	1	1
	Professional training					
	No	140	56 *(31%)*	84 *(27%)*	1.26 (0.82-1.91)	120 (0.77-1.87)
	Yes	355	123	232	1	
Health-related questions at baseline	Self-rated health					
	Bad	71	28 *(16%)*	43 *(14%)*	1.18 (0.68-2.03)	1.13 (0.60-2.13)
	Good	424	151	273	1	1
	PCS-SF12					
	< median (low)	241	79 *(45%)*	163 *(52%)*	0.75 (0.51-1.11)	0.80 (0.52-1.23)
	> median (high)	242	95	147	1	1
	MCS-SF12 (N = 483)					
	< median (low)	241	93 *(53%)*	148 *(48%)*	1.25 (0.85-1.84)	1.23 (0.84-1.96)
	> median (high)	242	81	161	1	1
	Number of problems at baseline (0–33)					
	Many problems (10+)	231	82 *(47%)*	149 *(48%)*	0.97 (0.66-1.43)	0.91 (0.59-1.38)
	Fewer problems (7–9)	254	92	162	1	1
	Medical contacts per year					
	>5	224	76 *(43%)*	148 *(47%)*	0.84 (0.57-1.24)	0.82 (0.54-1.24)
	≤5	269	102	167	1	1
	Smoking					
	Yes	265	102 *(59%)*	163 *(52%)*	1.29 (0.87-1.91)	1.47 (0.98-2.19)
	No	220	72	148	1	1
Factors related to the intervention study	Randomized toIntervention	240	88 *(49%)*	152 *(48%)*	1.04 (0.71-1.53)	0.97 (0.66-1.42)
	Control	255	91	164	1	1
	Number of included patients per physician					
	Few (≤13)	115	49 *(27%)*	66 *(21%)*	1.43 (0.91-2.23)	1.39 (0.88-2.18)
	Many (>13)	380	130	250	1	1

*Adjusted for the other variables in table.

Proportions and odds ratios (OR).

Health-related quality of life was not a significant predictor; however, low mental health (MCS-SF12) trended toward a correlation with lower response rate, and low physical health (PSC-SF12) trended toward a correlation with increased response. Factors such as education, professional training, civil status, and self-rated health were examined, but did not demonstrate any significant influence.

In order to illustrate whether the increased number of responders from 64% to 74% changed the outcome of the intervention after 1 year, we compared the improvement in selected variables from baseline to the 1-year follow-up regarding health, resources, and lifestyle. Table 2 compares the changes of these variables in the intervention and control groups among primary responders, responders after telephone reminding, and all responders. Overall, the additional responses did not change the estimates of the 1-year effect on health and lifestyle changes, but did increase the precision of the results. Although the numbers were small, responders after telephone reminding tended to profit more from the intervention than primary responders with respect to weight loss, smoking cessation, and alcohol habits (Table 2). Moreover, the observed improvement in MCS-SF12 in the intervention group was even greater in the group that responded after telephone reminding (4.2 versus 4.9).

**Table 2:** **Effect of telephone reminder at the 1 year follow-up on health and lifestyle outcomes**

	Total number = 364		Primary responders (N = 316)		Responders after telephone reminding (N = 48)		All responders (N = 364)	
			Intervention (N = 152)	Control (N = 164)	Intervention (N = 28)	Control (N = 20)	Intervention (N = 180)	Control (N = 184)
Physical health SF12-PCS (Mean score)	N = 353	1 year	48.0	48.8	49.9	44.7	48.2	48.3
		Baseline	47.0	47.5	48.6	46.1	47.2	47.3
		Improvement*	01.0	01.3	01.2	-1.4	01.0	01.0
		Difference** (95% CI)	-0.3 (-2.4 to 1.7) NS		2.6 (-2.2 to 7.4) NS		0.01 (-1.8 to 1.9) NS	
Mental Health SF12-MCS (Mean score)	N = 353	1 year	47.0	44.7	46.2	42.8	46.8	44.5
		Baseline	39.8	41.7	38.7	40.1	39.6	41.5
		Improvement*	07.2	03.0	07.6	02.7	07.3	03.0
		Difference** (95% CI)	*4.2 (1.3 to 7.0) P = 0.005*		4.9 (-2.8 to 12.5) NS		*4.3 (1.6 to 6.9) P = 0.002*	
Mean number of problems (range 0–33)	N = 364	1 year	8.3	9.3	8.2	9.9	8.2	9.3
		Baseline	10.2	10.2	9.2	9.6	10.0	10.1
		Improvement*	-1.9	-0.9	-1.0	0.3	-1.8	-0.8
		Difference** (95% CI)	*-1.0 (-1.9 to 0.05) P = 0.04*		-1.3 (-40.0 to 1.4) NS		*-1.0 (-1.8 to -0.2) P = 0.027*	
Mean self-rated health (range 0–5)	N = 355	1 year	2.6	2.5	2.4	3.1	2.6	2.6
		Baseline	2.8	2.6	2.5	2.8	2.8	2.7
		Improvement*	-0.2	-0.1	-0.1	0.3	-0.2	-0.1
		Difference** (95% CI)	-0.1 (-0.3 to 0.04) NS		0.04 (-0.06 to 0.8) NS		-0.15 (-0.3 to 0.008) P = 0.06 NS	
Mean weight loss when over weight (BMI > 25 at baseline), kg	N = 160	1 year	85.7	87.0	90.6	92.6	86.4	87.6
		Baseline	88.1	88.7	96.7	91.3	89.3	89.0
		Improvement	-2.4	-1.7	-6.1	1.3	-2.9	-1.5
		Difference** (95% CI)	-0.7 kg (-2.9 to 1.7) NS		-7.4 kg (-14.4 to 0.5) NS		-1.4 kg (-3.7 to 0.8) NS	
Eating more fiber	N = 364	1 year	93 (61%)	87 (53%)	18 (64%)	10 (50%)	111 (62%)	97 (53%)
		Difference**	8% (-3 to 18) NS		14% (-13 to 40) NS		8.7% (-1.5 to 19) NS	
Doing more exercise	N = 364	1 year	55 (36%)	46 (28%)	11 (39%)	6 (30%)	66 (37%0	52 (28%)
		Difference** (95% CI)	8% (-2.1 to 18) NS		9% (-18 to 33) NS		8.4% (-1.2 to 18) NS	
Smoked yesterday	N = 364	1 year	72 (47%)	91 (56%)	16 (57%)	13 (65%)	88 (48%)	106 (57%)
		Difference** (95% CI)	-9% (-20 to 1.7) NS		-8% (-33 to 19) NS		-8.7% (-18 to 1.5) NS	
Smoking less or quit tobacco	N = 352	1 year	28 (19%)	26 (16%)	7 (25%)	0	35 (20%)	26 (14%)
		Difference** (95% CI)	3% (-5 to 12) NS		*25% (4.7 to 43)*		6.4% (-1.8 to 14)	
Drinking less or quit alcohol	N = 364	1 year	22 (15%)	26 (17%)	9 (32%)	1 (5%)	31 (17%)	27 (15%)
		Difference** (95% CI)	-2.6% (-11 to 6) NS		*27% (3.7 to 46)*		2.5% (-5 to 10) NS	

NS, not significant.

*Improvement = 1 year - baseline (in SF12 positive) and improvement in problem is negative (fewer problems).

**Difference is between intervention and control.

## Discussion

A telephone reminder by a physician raised the response rate for a 1-year follow-up after preventive consultations for young adults with multiple psychosocial and lifestyle problems from 64% to 74%. Being a man was the only factor that significantly predicted non-response. Overall, the increased response rate did not change the estimates of the intervention’s effect on different health resources and lifestyle outcomes. However, the additional responders did increase the strength of the study. Responders after telephone reminding exhibited the same effect or a tendency toward a more beneficial effect of the intervention on mental health, number of problems, weight loss, smoking, and alcohol habits; however, the figures were small and must be interpreted with caution.

This study may have certain weaknesses. The data are derived from a survey conducted more than 10 years ago, and since then telecommunications has evolved considerably. Because of the wide use of mobile telephones, today people can be contacted whether or not they are at home. Our study population consisted of people with multiple psychosocial and lifestyle problems, which may limit the usefulness of these results in other populations. Owing to the number of patients, the study might have lacked power to identify significant associations. The only significant predictor for non-response was for males, and the difference in outcome between primary responders and all responders was only significant with respect to mental health. This study’s particular strength was the detailed baseline information from all participants, including non-responders, from questionnaires before the intervention.

The increase in response rate in the present study was at the same level as in other studies, regarding both the proportion of respondents that we were able to contact by telephone [14–17] and the 10% increase in the response rate [14–16, 18]. Among non-responders, participants aged 20 to 30 years and men were particularly overrepresented. The literature reveals different trends regarding non-response in relation to sex and age. Some reports have found, as we did, overrepresentation of young men among non-responders, while other studies have found no differences with respect to sex or age [14, 19–22]. Low mental health tended to predict non-response in this study, although there was no statistically significant correlation. This finding is supported by a study conducted in California in which those responding late or after a reminder by telephone were in mentally poorer health than those who responded early [14]. In relation to physical health, other studies - opposite ours - found an increased tendency toward non-response among study participants in bad physical health [23, 24]. A Danish register-based study demonstrated that non-response was associated with increased mortality [25], possibly indicating poor health among non-responders. The differences between the literature and our study might be explained by the low number of participants in our study, as well as the selected population.

The potential influence of non-responders on estimates has been investigated in several studies. In a cohort study of knee pain and osteoarthritis with an 18-month follow-up period, there was some evidence of selective non-participation but no significant bias in relation to the estimates concerning symptoms and clinical findings. Even though only few in the target population participated, the main effect of non-participation was a loss of precision in stratum-specific estimates [26]. Similar conclusions were drawn in a prospective cohort study, in which it was found that a prospective analysis in a cohort of relatively young, highly mobile, adult military personnel was not substantially biased by non-response at the first follow-up after four years [27].

In one survey that compared early and late responders, inclusion of the late responders did not change the health characteristic profile of the cohort [17]. It was suggested that instead of labor-intensive effort (e.g., telephone contact), consideration should be given to sending mail to a larger sample of the population and accepting a lower response rate [17]. The design of our study did not allow us to increase the number of questionnaires at follow-up because the number of participants was determined at baseline.

The consequences of declining participation rates in epidemiologic studies have been discussed in a recent review [28]. The authors concluded that a low participation rate does not necessarily indicate a high level of bias because it is the difference among responders and non-responders and the relation to exposure and outcome that determines whether bias is present [28]. A Danish study examined the effect of multiple reminders on non-response bias, prevalence estimates, and exposure-outcome relations. They concluded that raising the response rate with second and third mailings did not remove many of the differences between respondents and non-respondents, and was unlikely to eliminate non-response bias. They observed only small changes in exposure-outcome relationships after raising the response rate [8]. The findings that increased response rates after reminders had only minor effects on response patterns and study conclusions have been described in other settings [9–11], and are in agreement with the results of our study. Increasing the response rate with respondents that are not representative for all non-respondents might actually introduce more bias [28]. Including responders after telephone reminding in our study did not appear to introduce more bias into the study because it did not change the interpretation of the intervention study. However, this finding does not rule out the risk of selection bias, because non-responders are probably an inhomogeneous group. This assertion is supported by a recent study in which two distinct groups of non-participants were identified in a population-based breast cancer program [22]. Thus, a raised response rate contributes to increased statistical power, but will not necessarily reduce eventual selection bias.

## Conclusion

In this study, we found that a telephone reminder for a 1-year follow-up questionnaire among young adults with multiple psychosocial and lifestyle problems increased the response rate for returning questionnaires by mail of approximately 10%. Being a male predicted non-response. Inclusion of responders after telephone reminding at the 1-year follow-up did not change the estimates regarding the effect of the intervention study. However, the additional responses did contribute to the greater strength and precision of the results.
